# Elongated-Hexagonal Photonic Crystal for Buffering, Sensing, and Modulation

**DOI:** 10.3390/nano11030809

**Published:** 2021-03-22

**Authors:** Sayed Elshahat, Israa Abood, Zixian Liang, Jihong Pei, Zhengbiao Ouyang

**Affiliations:** 1 Institute of Microscale Optoelectronics, Shenzhen University, Shenzhen 518060, China; selshahat@szu.edu.cn (S.E.); zixianliang@szu.edu.cn (Z.L.); 2 College of Electronics and Information Technology, Shenzhen University, Shenzhen 518060, China; jhpei@szu.edu.cn; 3 THz Technical Research Center of Shenzhen University, Shenzhen Key Laboratory of Micro-Nano Photonic Information Technology, Key Laboratory of Optoelectronic Devices and Systems of Ministry of Education and Guangdong Province, College of Physics and Optoelectronic Engineering, Shenzhen University, Shenzhen 518060, China; i.abood@szu.edu.cn; 4 Physics Department, Faculty of Science, Assiut University, Assiut 71516, Egypt

**Keywords:** photonic crystal waveguide, optical buffers, sensors, dynamic modulation

## Abstract

A paradigm for high buffering performance with an essential fulfillment for sensing and modulation was set forth. Through substituting the fundamental two rows of air holes in an elongated hexagonal photonic crystal (E-PhC) by one row of the triangular gaps, the EPCW is molded to form an irregular waveguide. By properly adjusting the triangle dimension solitary, we fulfilled the lowest favorable value of the physical-size of each stored bit by about 5.5510 μm. Besides, the EPCW is highly sensitive to refractive index (RI) perturbation attributed to the medium through infiltrating the triangular gaps inside the EPCW by microfluid with high RI sensitivity of about 379.87 nm/RIU. Furthermore, dynamic modulation can be achieved by applying external voltage and high electro-optical (EO) sensitivity is obtained of about 748.407 nm/RIU. The higher sensitivity is attributable to strong optical confinement in the waveguide region and enhanced light-matter interaction in the region of the microfluid triangular gaps inside the EPCW and conventional gaps (air holes). The EPCW structure enhances the interaction between the light and the sensing medium.

## 1. Introduction

Slotted photonic crystal waveguides (SPCWs) with various PCW modalities, have been anticipated theoretically and experimentally demonstrated [1]. They are created by opening a narrow slot or irregular air gaps as a line-defect inside a PCW. Due to the discontinuity at the interface of silicon slab with high refractive index and air gaps with low index, the electric/magnetic field in the air gaps with low index is enhanced [2], with associated optical-buffer enhancement accessible from the PCW [3]. They can be advantageously used in high-sensitivity sensors [4], optical switches [5], and high-speed electro-optical modulators [6], etc. Optical buffers can store data temporally and adjust the timing of optical packets [7]. Optical buffer is a significant component for all-optical communication networks, processors, and optical computers in the future. The unique PCW nanostructure designed is efficient for optical buffering performances [3,8] because of room-temperature operation [9], masterful manipulation of the guided-mode dispersion relations with a change in subtle structure parameters, and compatibility for on-chip integration [10].

Besides, by fine-tuning the defects in a PCW or infiltrating appropriate materials, photonic crystal (PhC) devices are truly auspicious for sensing attributable to the bright features like high sensitivity, ultra-compact size, structural design flexibility [11,12], and suitable for massive integration [13]. Particularly, air-gap infiltration with microfluids in the PCWs has been suggestively demonstrated [14] that can make availability variation in microfluid refractive index, which enhances the dispersion and sensing properties of PCWs [15].

In this paper, the proposed structure is based on the merging between hexagonal and square lattices to produce an elongated hexagonal photonic crystal structure (E-PhC). Through substituting the central two rows of air holes with one row of triangular gaps, an irregular waveguide is designed. The triangular gaps inside the proposed structure behave as microcavities of a low-refractive-index. At this point, every triangle can be considered a defect and every defect can be considered as a cavity. Thus, a sequence of cavities can couple together to form a line of cavities or the defect-cavity waveguide. Firstly, by properly adjusting the triangular dimension, high buffering performance has been attained. Then, due to the strong light confinement, which gives intensification to the optical mode with a guided-mode wavelength, the sensitivity to the refractive index (RI) through infiltrating the air gaps by microfluid was studied. Finally, dynamic modulation with measuring electro-optical (EO) sensitivity based on the proposed EPCW can be achieved by filling the EO-effect polymer into the air gaps and holes. To sum up, the enhanced structure is vastly appropriate for optical buffering and is completely possible due to the limited optimization process.

## 2. Structure Model and Results

**Figure 1:**
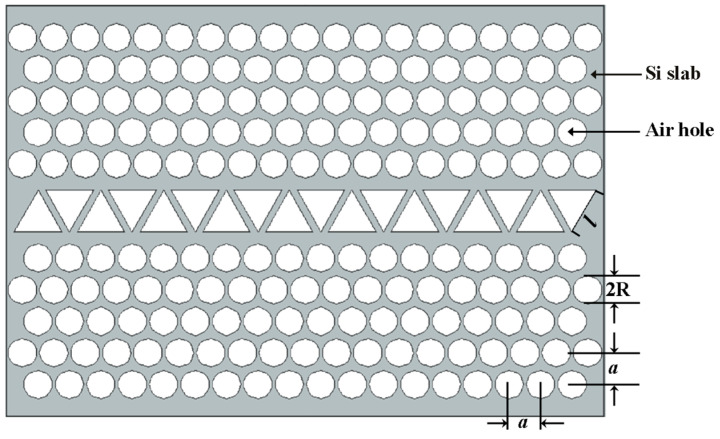
Schematic diagram of the proposed EPCW structure.

**Figure 2:**
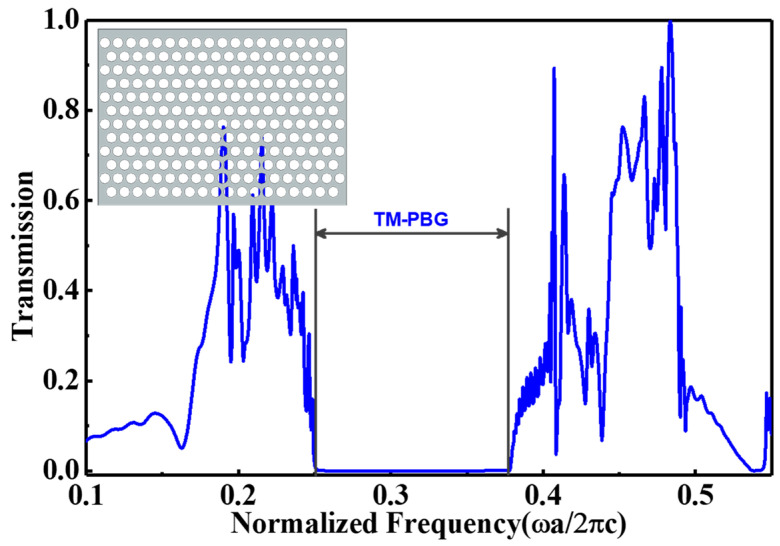
The transmission spectrum of the basic E-PhC slab of n = 3.48 and R = 0.45a.

The scrutinized structure is based on the merging between hexagonal and square lattices to produce E-PhC [16]. The constructed structure is the PhC slab of n=3.48 with fixed and large air holes of radius R = 0.45a, where a is the lattice period. The proposed EPCW is created through substituting two rows of fundamental air holes by one row of equilateral triangular gaps inside the EPCW to form an irregular waveguide. Each triangle has a side length l as shown in Figure 1. By properly adjusting the triangle dimension solely without varying the lattice constants, R and a, high buffering performance with an essential performance for sensing and modulation have been attained. Suppose, for instance, that the dielectric constant is independent on the *Y*-axis. Then the solutions all take the form of either transverse electric (TE) modes with nonzero $\left(H_{x},\;H_{z},E_{y}\right)$ or transverse magnetic (TM) modes with nonzero $\left(H_{y},\;E_{x},\;E_{z}\right).$ For clarity, firstly, the photonic band gaps (PBGs) have been simulated for TM by the spectral features of the basic E-PhC structure of a silicon slab at n = 3.48 and R = 0.45a as shown in the inset of Figure 2. Via a fast Fourier transform (FFT) algorithm, the output of the transmission spectrum in the frequency domain from the time domain is shown in Figure 2. The transmission spectrum has a large TM PBG of normalized frequency from ωa/2πc =0.2485 to 0.3805 along the propagation direction Γ–X.

**Figure 3:**
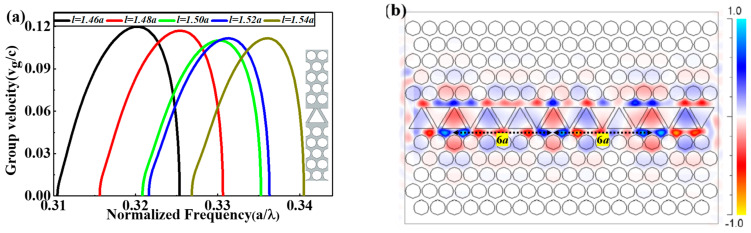
(**a**) Group velocity $\left(v_{g}/c\right)$ for TM guided-mode with the normalized frequency U = ωa/2πc = a/λ  at different values of l, whereas the inset figure shows the supercell model for PWE calculations; (**b**) the M-field profile of the transmission pulse at l = 1.50a.

The EPCW has a perfect buffering performance in a wide-range variation of triangle defect length l. Whilst, we will concern about the variation of l in the range from 1.46a to 1.54a with an increment of Δl=0.02a for high optical buffering properties. Figure 3 explains the variation of the group velocity $\left(v_{g}/c\right)$ for TM guided-mode with the normalized frequency U=ωa/2πc=a/λ via the plane wave expansion (PWE) method through the module BandSOLVE of RSoft software, whereas the inset figure shows the supercell model for PWE calculations. It can be seen from Figure 3a that the group velocity decreases from high values until l = 1.50a and then starts to increase once more with the curve moving to a higher frequency area. The transmission mechanism of TM light pulse inside the proposed EPCW can be understood by the magnetic field (M-field) profile shown in Figure 3b. The M-field of the transmission pulse is highly confined around and inside the triangular gaps of EPCW and different from that observed in conventional PCWs. We observe spatially light confinement and periodic oscillations in the field profile lengthwise on the propagation path, displaying a strong coupling between the triangular defect cavities that form a channel waveguide. The M-field profile shows the pulse being in periodic alternating between compression and expanding, in which the hop between each two neighboring spatial compressions is 6*a* as shown in Figure 3b. Wherefore, the EPCW may be used to generate vastly low-threshold lasers, in optical modulators and can be used for enhancing dealings between light and sensing media.

Combined with the transmission determination in data interconnections, the EPCW is superior for realizing all-optical buffers in practical applications for its compact physical size, owing to its performance of delaying light in optoelectronic devices. The ingoing data into the buffer possesses a categorization of packets (i.e., sequences of bits) with the optical central wavelength $\;\lambda_{0}$. The data bit period in each packet is $\tau_{bit}$ with a base-bandwidth $\;B_{packet}=1/\tau_{bit}$. The optical data bandwidth is $∆\overset{’}{\omega }\;=\;4\pi \;B_{packet}$ [17] in rad/s and can be defined through the normalized bandwidth by $∆U\;=\;\left(a/2\pi c\right)∆\overset{’}{\omega }$ [18]. The key factor for all delay-line optical buffers is the physical size of each stored bit in the buffer $L_{bit}={\bar{v}}_{g}\times \tau_{bit}\;=\;{\bar{v}}_{g}\times \left(2a/c∆U\right)$ [17], which is independent of the delay line length. To minimize the physical size of each stored bit or maximize the number of stored bits in the buffer, a low group velocity compatible with wider transmission bandwidth is required.

The simulation data in Figure 3 is presented in Table 1 for clarity. The vital wavelength can be scrambled to be 1.55 μm via contraction the lattice period *a* from 1 μm to the values according to Table 1. It is obvious from them that the optimum case of the proposed EPCW is at l = 1.50a which shows a wider bandwidth of about 56.6128 nm with ${\bar{v}}_{g}\;=\;0.0654c$ within ±10% variation of $v_{g}$ corresponding to the lowest $L_{bit}\;=\;5.5510\;\mu m$.

Consequently, in that case for storing 1 bit, a storage length of 5.5510 μm is required. In comparison with other previous structures, for example in the case of photonic crystal coupled-cavity of micro-optical buffer, the lowest physical size of each stored bit $L_{bit}$ reached 16.33 μm [18]. After a series of modification in silicon-polymer photonic-crystal coupled-cavity waveguide $L_{bit}$ was 7.97 μm [19], and by introducing air holes in W2 PCW $L_{bit}$ was 10.85 μm [15]. Mainly for all new-generation communication systems and optical signal processing, high bit rates are needed with a small storage length. The EPCW is a potential candidate for all the requirements of optical-signal processing. Thus, we can say that introducing triangular defects in EPCW possesses an advantage for enhancing the coupling between the defects to enlarge bandwidth, group delay, and low $L_{bit}$.

## 3. Refractive-Index Sensing

**Figure 4:**
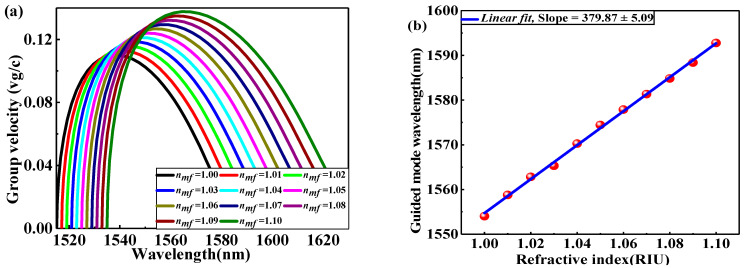
(**a**) Group velocity $v_{g}/c$ as a function of wavelength due to changing RI of $n_{mf}$ from 1.00 to 1.10 with an increment of $\Delta n_{mf}\;=\;0.01$, (**b**) the selected guided mode wavelength for each value of $n_{mf}$.

As mentioned above, the EPCW exhibits strong light confinement, which gives intensification to the optical mode with a guided-mode wavelength that is vastly sensitive to refractive index (RI) perturbation attributed to the medium through infiltrating the air holes of EPCW by microfluid. RI sensor was established based on the optimum EPCW of l fixed at 1.50a and for operating at the optical communication wavelength, the lattice constant was selected to be a = 0.5084 μm. Figure 4a shows the variation of $v_{g}/c$ with the operating wavelength due to filling the triangular gaps inside the EPCW by microfluid with changing microfluid refractive index of $n_{mf}$ from 1.00 to 1.10 with an increment of $\Delta n_{mf}\;=\;0.01$ as shown in Figure 4a, all guide-mode wavelengths are deflected towards the higher wavelength (i.e., redshift) as $n_{mf}$ increases. When $n_{mf}$ changes from 1.00 to 1.10, ${\bar{v}}_{g}$ changes from 0.085c to 0.110c with the shifting of the central guide-mode wavelength by nm from 1554 to 1591. In designed sensor devices, sensitivity (*S*) is the imperative indicator for appraising sensing ability. S measures the variation of the guided-mode wavelength ∆λ produced during the change in refractive index $∆n_{mf}$ of the microfluid filled in the triangular gaps inside the EPCW and can be expressed via the relation $S\;=\;∆\lambda /∆n_{mf}$, by the unit nm/RIU. To distinguish the S property of the proposed EPCW. Whereas, each value of $n_{mf}$ has its own curve of $v_{g\;}$ variation with wavelength as shown in Figure 4a. The corresponding wavelength to average group velocity within ±10% variation of $v_{g}$ or $n_{g}$ and most of the practical applications in this range [20] is the guided-mode wavelength. The selected guided-mode wavelengths corresponding to each value $n_{mf}$ is shown in Figure 4b. The simulated data is characterized by the red sphere, and the linear fitting by the blue solid line. From linear fitting, S, the slope is 379.87 nm/RIU, we accomplished a high value of RI sensitivity in the EPCW as compared with most previous studies based on the conventional PCW as shown in Table 2. This high sensitivity is due to the strong optical confinement and light-matter interaction in the region of the slots, the EPCW structure can increase the interaction region between light and the sensing medium.

The group indices $\left(n_{g}\;=\;c/v_{g}\right)$ can be evaluated experimentally via Fourier transform spectral interferometry as a function of wavelength when the proposed structure is placed in one arm of an unbalanced Mach–Zehnder interferometer [21]. The setups for both measurements of the transmission and group index are shown in detail in [22]. Moreover, by using an optical network analyzer (Q7750, Advantest), the transmission and group index spectra can be measured directly through the modulation phase-shift method according to [23,24].

**Table 2:** Comparison of RI sensitivity in EPCW with previous studies based on the conventional PCW.

Structure	S (nm/RIU)
EPCW	379.87
SDPCW [15]	244
Nano-slotted micro-ring resonator [25]	100
PCW in SOI [26]	174.8
SOI ring-shaped PCW [27]	66
Ring-shaped PhC [28]	110

## 4. Dynamic Modulation and Electro-Optical Sensor

**Figure 5:**
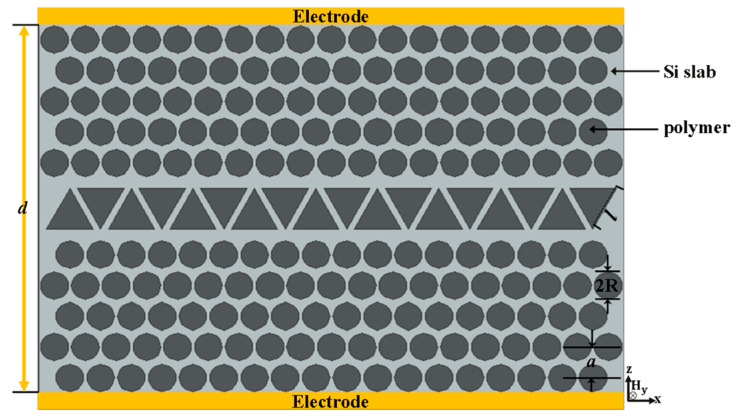
Schematic conformation of the dynamic modulation for the optimum EPCW R = 0.45a, l = 1.50a and a = 0.5084 μm.

It is outstanding to realize that the physical characteristics of transmission required can be controlled through an external command to implement optical buffers applications. Moreover, to understand the dynamic modulation of optical-buffer performances in the proposed EPCW, two electrodes are set on both sides of the optimum EPCW of l fixed at 1.50a, and for operating at the optical communication wavelength, the lattice constant was selected to be a = 0.5084 μm with R = 0.45a as shown in Figure 5. Then the electrostatic field lines are parallel to the *Z*-axis, to allow the largest linear electro-optic coefficient $\gamma_{33}$ in polystyrene [29]. In this context, our electro-optical (EO) sensor based on the proposed EPCW can be achieved by filling the EO-effect polymer into the air gaps (triangles and holes).

Particularly, air holes infiltrated with liquid in PCWs have been significantly investigated and demonstrated [14]. Different microfabrication methods have been applied in the polymer-based micro-optical device technology, including soft-lithography, electron-beam-lithography (EBL) [30], and electron beam etching [31]. The fabrication conditions should be chosen carefully to diminish the fabrication errors of the designed parameters. Consequently, it is essential to use some processes with high accuracies, such as high-resolution lithography and high-quality pattern. Thus, the method of direct writing by an electron beam is particularly attractive to obtain high-resolution without using further etching steps or photolithography masks [32,33]. On the other hand, nanoimprint lithography (NIL) can be used to emboss stamps with high aspect ratio lines and rod arrays into thin polymer films directly and to transfer these patterns into several materials [34], while NIL is regarded as a developing technique for next-generation lithography because of their producibility of nano-scale features [35]. Therefore, the NIL process could be appropriate for the fabrication of polymeric PhC structures [36]. For example, A silicon/organic hybrid modulator integrating PCW, 75 nm slot, EO polymer infiltration is experimentally demonstrated [37] and also, high-speed silicon modulator with a low-driving voltage based on a polymer infiltrated slow-light PCW is demonstrated [6].

By applying an external modulation voltage, the polymer refractive index will be changed based on the Pockels effect. The variation is subject to the second-order susceptibility $\chi^{〈2〉}$ [38] and can be calculated as $∆n\;=\;-\;0.5\;n_{poly}^{3}\;\gamma_{33}V/d$, where $\gamma_{33}$ is the linear electro-optic coefficient, $∆n_{poly}$ represents the extraordinary refractive index of polymer, V is the applied modulating voltage, d is the distance between the two electrodes, where $\gamma_{33}$ should be by μm/V at the time, V by V, and d by μm so V/d by V/μm. The nonlinear effects can be significantly enhanced in the proposed EPCW with low group velocity, which is due to the local density compression of states produced by the slow light. In view of the effect on the second-order susceptibility induced by the nanostructure, the effective second-order susceptibility in the EPCW depends on the local field factor f and the polymer bulk susceptibility $\chi_{bulk}^{〈2〉}$ [38], thus $\chi_{pc}^{〈2〉}\;=\;f^{3}\chi_{bulk}^{〈2〉}$. In that case, the electro-optic coefficient converts to be $\gamma_{33}f^{3}$ and the local field factor f is calculated with the group velocity inside the bulk polymer substrate $\nu_{g}^{bulk}\;=\;c/n_{ploy}$, and the group velocity in PhC structure $\nu_{g}^{pc}$. The f in EPCW can be calculated as $f\;=\;\sqrt{\nu_{g}^{bulk}/\nu_{g}^{PC}}$. The modified index variation can be expressed as [29]: $∆n\;=\;n_{p}\left(V\right)-n_{p}\;=\;-0.5{\left(n_{p}\times n_{g}\right)}^{\frac{3}{2}}\gamma_{33}V/d.$ Then the dynamic modulation in the optimized EPCW with R = 0.45a, l = 1.50a and a = 0.5084 μm is investigated with $\gamma_{33}\;=\;150\;\mathrm{pm}/V$ [6], $n_{p}\;=\;1.59,\;n_{g}\;=\;15.29$ and d = 12a = 6.1008 μm.

**Figure 6:**
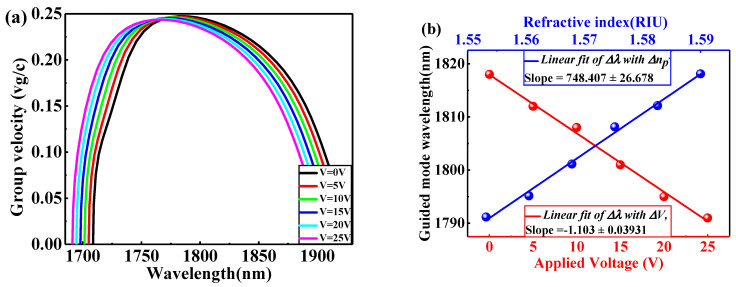
(**a**) The variation of $v_{g}/c$ with the wavelength due to different applied modulation voltages from 0 V to 25 V with an increment of ΔV = 5 V based on filling the air gaps (triangles and holes) with the nonlinear optical (NLO) polymer; (**b**) the selected guided mode wavelengths corresponding to each value of V and $n_{p}$.

By fine-tuning the external modulated voltage on the electrodes, the optical buffering performance and EO sensing are investigated by adjusting V from 0 V to 25 V with an increment of ΔV = 5 V. Figure 6a shows the variation of $v_{g}/c$ with the operating wavelength due to different applied modulation voltages based on filling the air gaps (triangles and holes) with the nonlinear optical (NLO) polymer of $n_{p}\;=\;1.59$ at V = 0 V. As shown in Figure 6a, all guide-mode wavelengths are deflected towards the lower wavelength (i.e., blueshift) as the $n_{p}\left(V\right)$ decreases due to increasing the applied modulated voltage. When V changes from 0 V to 25 V with an increment of ΔV = 5 V, which corresponds to a change in polymer refractive index from 1.59 (0 V) to 1.55316 (25 V) with an increment of $\Delta n_{p\;}\left(5\;V\right)=\;-0.00737$, with a shifting of the central guide-mode wavelength by nm from 1818 (0 V) to 1791 (25 V) as shown from Figure 6a. By the way, we mentioned in the previous section that S measures the shift of the guided-mode wavelength ∆λ produced during the change in refractive index, so it can be called as RI sensitivity. At this time for analyzing the sensitivity ($S_{v}$) of the EPCW EO sensor, the shift of the guided-mode wavelength ∆λ is observed as a function of the change in the applied modulated voltage ∆V and can be expressed as:

$$S\;=\;\frac{∆\lambda }{∆V}\;by\;\left(\frac{\mathrm{nm}}{V}\right)\mathrm{unit}\;=\frac{∆\lambda }{∆n_{p}}\times \frac{∆n_{p}}{∆V},\;S_{v}=S\times \frac{\Delta n_{p}}{\Delta V},$$

which can be called the voltage sensitivity. Each value of V and $n_{p}$ has its own curve of variation with wavelength as shown in Figure 6a. The corresponding wavelength to average group velocity is the guided mode wavelength. The selected guided-mode wavelengths corresponding to each value of V and $n_{p}$ are shown in Figure 6b. The simulated data and the linear fitting are characterized by the spheres and solid lines, respectively.

From the linear fitting of Figure 6b, the voltage sensitivity $S_{v}$, the slope, is 1.103 nm/V. Besides, the RI sensitivity can be calculated as 748.407 nm/RIU. Compared to a lattice-shifted resonant microcavity in a triangular lattice PhC slab in [39], $S_{v}$ is 31.90 nm/V and corresponds to a refractive index changes ∆n = 0.001 results in a ∆λ = 0.18 nm (RI sensitivity is ~180 nm/RIU). The slotted PCW-based EO modulator demonstrated by [40], where the ∆λ is 0.12 nm as refractive index change of ∆n = 0.001, (RI sensitivity is ~120 nm/RIU). Also, polymer-filled PCW demonstrated by [41], the operating wavelength changes from 1542.02 nm (0 V) to 1533.31 nm (120 V), ~9 nm/120 V ($S_{v}$ sensitivity is only 0.0725 nm/V). The proposed structure presented in this paper is preferable as compared to the previous work. The higher sensitivity is attributable to strong optical concentration in the waveguide region and enhanced light-matter interaction in the region of the microfluid triangular gaps inside the EPCW and conventional gaps (air holes), i.e., the EPCW structure enhances the interaction between the light and sensing medium.

**Figure 7:**
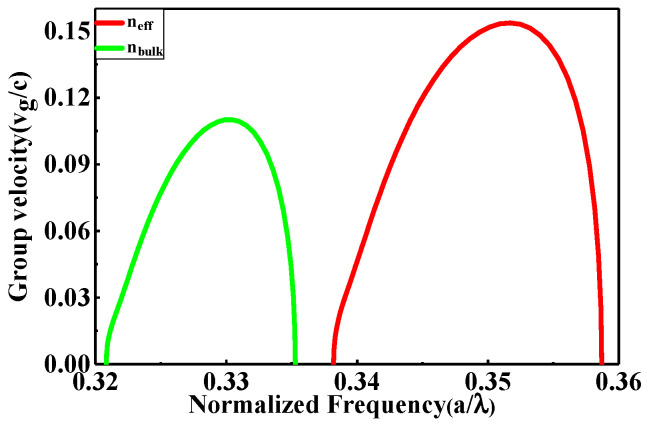
Group velocity $\left(v_{g}/c\right)$ for TM guided mode with the normalized frequency U = ωa/2πc=a/λ  for 2-D calculations with $n_{eff}\;=\;3.2$ approximation and $n_{bulk}\;=\;3.48\;$ of the optimized mode at l = 1.50a.

To strictly validate the simulation results, 3-D simulations are required. However, 3-D calculations are enormously time-consuming [23,42]. Fortunately, it was demonstrated that 2-D calculations of PhC structures with the effective refractive index $n_{eff}$ with infinite height is a good approximation for 3-D structures, which is frequently treated by researchers and appropriated experimentally [23,43]. The 2-D calculations are generally applied with $n_{eff}\;=\;3.2$ [16,43,44,45,46] in place of slab bulk refractive index. Figure 7 compares the group velocity $\left(v_{g}/c\right)$ for TM guided-mode via the normalized frequency U=ωa/2πc=a/λ for 2-D calculations with $n_{eff}\;=\;3.2$ approximation and $n_{bulk}\;=\;3.48$ for the optimum case of the proposed EPCW at l = 1.50a. Figure 7 shows that for $n_{bulk}\;=\;3.48$ it possesses a narrow waveband with lower group velocity, on the contrary $n_{ff}\;=\;3.2$ it possesses a wider waveband but with the higher group velocity. The waveband ranging from 0.3396(2πc/a) to 0.3573(2πc/a), under the ±10% variation of $n_{g}$ for 2-D calculations of $n_{eff}=3.2$, corresponds to wider-bandwidth about 78.3781 nm with $L_{bit}\;=\;5.7726\;\mu m$. Whileas, for 2-D calculations of $n_{bulk}\;=\;3.48$, the waveband is from 0.3220 (2πc/a) to 0.3340 (2πc/a), which corresponds to narrow-bandwidth ∆λ about 56.6128 nm with $L_{bit}\;=\;5.5510\;\mu m$. It can be seen that the results for the two cases are approximately equal. The curves in the two cases can be explained as: from the common equation (c/n =ω/k), with the increase of refractive index, the slow light bands, $v_{g}$ curves move toward the lower frequency area, as *k* being unchanged. As well, from the theorem of electromagnetic variation [47]: a higher refractive-index region would lead to a lower mode frequency. Therefore, the $n_{eff}$ approximation leads to the higher average group velocity. Mostly, the properties of PhC can keep on unchanged, providing one expands or contracts the parameters of PCW structures proportionally. The value of the optimum operating wavelength can be selected of the structure by only setting the value of a without simulation repetition.

## 5. Conclusions

In conclusion, a new type of defect is introduced which is created by substituting the fundamental two rows of air holes by one row of the triangular gaps inside the EPCW to form an irregular waveguide. By properly adjusting the triangle dimension solitarily without varying the lattice constant, R and a, high buffering performance with an essential performance for sensing and modulation have been attained. Lower physical size of 5.5510 μm for each stored bit is achieved. Besides, the EPCW can be used as a RI sensor with a RI sensitivity of 379.87 nm/RIU by filling the triangular gaps inside the EPCW with microfluid for a changing microfluid refractive index of $n_{mf}$ from 1.00 to 1.10 with an increment of $\Delta n_{mf}\;=\;0.01$. We accomplished a high value of RI sensitivity in the EPCW as compared with most of previous studies based on conventional PCWs. Besides, the EPCW was studied as EO voltage sensor by fine-tuning an external modulated voltage V from 0 V to 25 V for the air gaps (triangles and holes) filled with the nonlinear optical (NLO) polymer. The high sensitivity of EO voltage sensor is obtained as 1.103 nm/V, which corresponds to a RI of 748.407 nm/RIU.

## Figures and Tables

**Table 1:** EPCW optical buffering properties at different values of triangle-defect side length *l*.

Parameter (l/a)	∆U	∆λ at 1550 nm	a (μm)	${\bar{v}}_{g}/c$	$L_{bit}\;\left(\mu m\right)\;$
1.46	0.0113	55.2527	0.4931	0.0887	7.7164
1.48	0.0118	56.3811	0.5011	0.0833	7.1020
1.50	0.0120	56.6128	0.5084	0.0654	5.5510
1.52	0.0118	55.3925	0.5101	0.0702	6.0916
1.54	0.0105	48.6741	0.5173	0.0708	6.9864
